# Ga-doped ZnO transparent electrodes with TiO_2 _blocking layer/nanoparticles for dye-sensitized solar cells

**DOI:** 10.1186/1556-276X-7-11

**Published:** 2012-01-05

**Authors:** Ji-Hong Kim, Kyung-Ju Lee, Ji-Hyung Roh, Sang-Woo Song, Jae-Ho Park, In-Hyung Yer, Byung-Moo Moon

**Affiliations:** 1 Department of Electrical Engineering, Korea University, 5-1 Anam-dong, Seongbuk-gu, Seoul, 136-713, South Korea

## Abstract

Ga-doped ZnO [GZO] thin films were employed for the transparent electrodes in dye-sensitized solar cells [DSSCs]. The electrical property of the deposited GZO films was as good as that of commercially used fluorine-doped tin oxide [FTO]. In order to protect the GZO and enhance the photovoltaic properties, a TiO_2 _blocking layer was deposited on the GZO surface. Then, TiO_2 _nanoparticles were coated on the blocking layer, and dye was attached for the fabrication of DSSCs. The fabricated DSSCs with the GZO/TiO_2 _glasses showed an enhanced conversion efficiency of 4.02% compared to the devices with the normal GZO glasses (3.36%). Furthermore, they showed better characteristics even than those using the FTO glasses, which can be attributed to the reduced charge recombination and series resistance.

## Introduction

Dye-sensitized solar cells [DSSCs] have been recognized as an alternative to the conventional p-n junction solar cells because of their simple fabrication process, low production cost, and transparency. A typical DSSC consists of a transparent conductive oxide [TCO] electrode, a dye-sensitized oxide semiconductor nanoparticle layer, a liquid redox electrolyte, and a Pt counter electrode [1,2]. Generally, fluorine-doped tin oxide [FTO] is commonly used for DSSCs as TCO due to its good thermal stability. However, FTO films have some drawbacks including high cost, insufficient conductivity, and low optical transmittance. Therefore, new TCO materials are required to replace FTO glasses [3].

ZnO-based materials have emerged as a promising material for transparent electrodes in solar cell applications. Since undoped ZnO shows high resistivity owing to low carrier concentration, group-III elements are doped into ZnO. Among them, Ga-doped ZnO [GZO] has several advantages such as higher resistance to oxidation and less lattice deformation compared to the other materials [4,5]. Nevertheless, little effort has been spent on attempts to use GZO as TCO for DSSCs since the surface structure of the ZnO-based materials may be destroyed when they are immersed in the acidic dye solution containing a Ru complex for a long time. Besides, the electrical conductivity of GZO films can deteriorate after thermal annealing at high temperature which is required to form the TiO_2 _semiconductor nanoparticle layer [6].

In this paper, we suggest the use of GZO transparent electrodes with a TiO_2 _blocking layer for DSSCs. The TiO_2 _blocking layer can protect the GZO electrodes from the acidic dye solution and the oxidation at high temperature. The use of a thin TiO_2 _blocking layer can also reduce the recombination of electrons at the electrode/electrolyte interface.

### Experimental details

GZO thin films were deposited on glass substrates by using a pulsed laser deposition [PLD] system for transparent electrodes. A ZnO ceramic target containing 2 wt.% Ga_2_O_3 _was ablated using a Q-switched Nd:YAG laser with a wavelength of 355 nm (Surelite III; Continuum, Santa Clara, CA, USA). During the deposition, oxygen partial pressure and substrate temperature were kept at the optimal conditions, which were 20 mTorr and 400°C, respectively. The electrical properties of the deposited GZO thin films were investigated by the van der Pauw Hall measurement system. After the deposition of the GZO films, a thin TiO_2 _layer was also deposited on the GZO surface for the blocking layer by a PLD method. A metal Ti target was ablated in oxygen atmosphere at a substrate temperature of 400°C. The structural and optical properties were examined by X-ray diffraction [XRD] and UV-Vis spectroscopy.

Using the fabricated GZO glasses, DSSCs were manufactured. Anatase TiO_2 _slurry (colloid) was prepared with a conventional method [7] and coated for a TiO_2 _nanoparticle layer. The coating was carried out on the TiO_2 _blocking layer with an active area of 0.36 cm^2 ^by doctor blading. For comparison, the GZO without the blocking layer and commercially used FTO glasses were also employed to fabricate the DSSCs. After being sintered at 510°C, they were immersed into the solution of N719 ruthenium-based dye. Pt-coated counter electrodes were prepared by screen printing on the GZO-deposited glasses and heated at 400°C. The surface morphologies of the TiO_2 _nanoparticles on the GZO and GZO/TiO_2 _glasses were observed by scanning electron microscopy [SEM]. Then, the DSSC cells were assembled by sealing the TiO_2 _electrode and Pt electrode together using a hot melt film (Bynel; DuPont, Wilmington, DE, USA), and an electrolyte was injected into the space between the electrodes through a predrilled hole in the counter electrode. Finally, the hole was sealed with Bynel and a cover glass. The photovoltaic performance of the DSSCs was evaluated using a solar simulator at one sun (AM1.5, 100 mWcm^-2^) condition. In order to investigate the electron transport properties of the DSSCs, electrochemical impedance spectroscopy analysis was performed.

## Results and discussion

**Figure 1:**
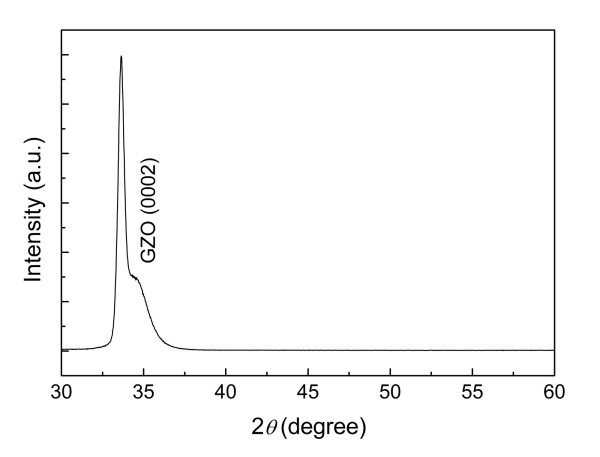
**XRD *θ*-2*θ *pattern of the GZO thin films deposited on glass substrates**.

Figure 1 shows the XRD *θ*-2*θ *spectra of the GZO thin films on glass substrates. The diffraction peak of GZO (0002) appears at 2*θ *= 33.64°, which means that the *c*-axis oriented GZO films were grown on glass substrates. GZO thin films are well known to have a hexagonal wurtzite structure with a preferred growth orientation along the *c*-axis due to their lowest surface free energy. The GZO films on glass showed a very low resistivity of 5.95 × 10^-4 ^Ω cm (sheet resistance of 6.53 Ω/sq), which is comparable to that of the commercially used FTO glasses. The carrier concentration and Hall mobility of the GZO films were 6.82 × 10^20^/cm^3 ^and 15.37 cm^2^/Vs, respectively. The high carrier concentration of the GZO films can be attributed to the substitution of Ga^3+ ^for Zn^2+ ^caused by the supply of sufficient thermal energy [8]. Furthermore, the deposition at the optimal substrate temperature leads to high mobility with the improvement in crystallinity. From these results, it is concluded that the deposited GZO films can be used for transparent electrodes. However, as mentioned above, a blocking layer should be needed in DSSC applications because of susceptibility to acid and oxidation at high temperature.

**Figure 2:**
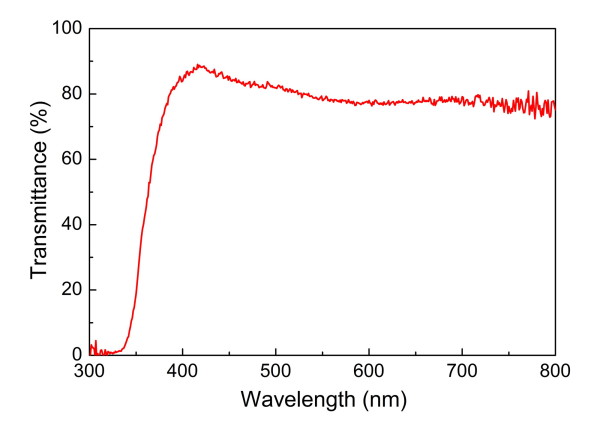
**Optical transmittance spectra for the GZO glasses with the TiO_2 _blocking layer**.

Figure 2 shows the optical transmittance spectra for the GZO glasses with the TiO_2 _blocking layer. The average transmittance is approximately 80% in the visible region, which implies that the fabricated GZO glasses are highly transparent enough to be applied to the DSSCs even after the deposition of the TiO_2 _blocking layer.

**Figure 3:**
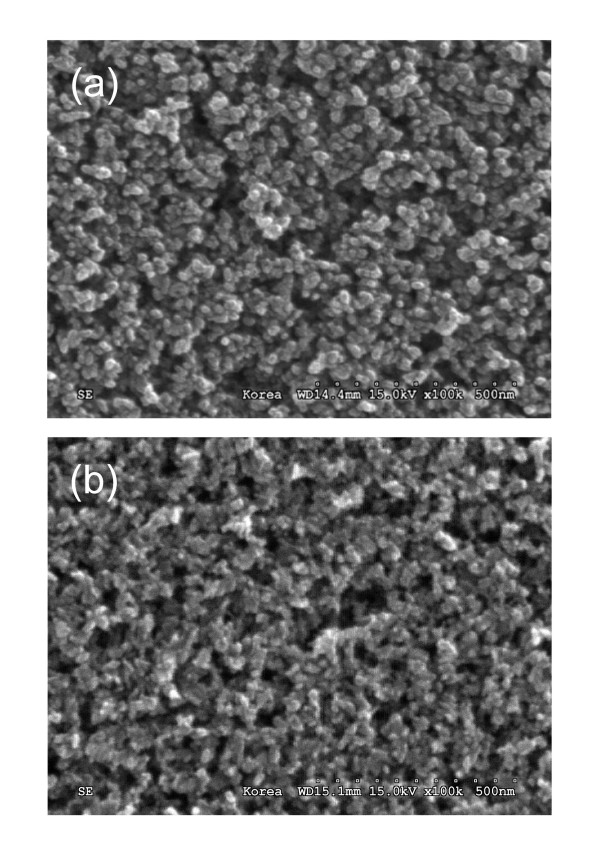
**SEM images of the TiO_2 _nanoparticles**. The TiO_2 _nanoparticles were coated on the (**a**) GZO glasses with the TiO_2 _blocking layer and (**b**) normal GZO glasses.

Figures 3a and 3b show the SEM images of the TiO_2 _nanoparticles coated on the GZO glasses with the TiO_2 _blocking layer and normal GZO glasses, respectively. It can be seen that the porous TiO_2 _nanoparticles adhere uniformly on both glasses. There is no large difference between them, but the morphology of the TiO_2 _nanoparticles on the TiO_2 _blocking layer is more ordered and spherical.

**Figure 4:**
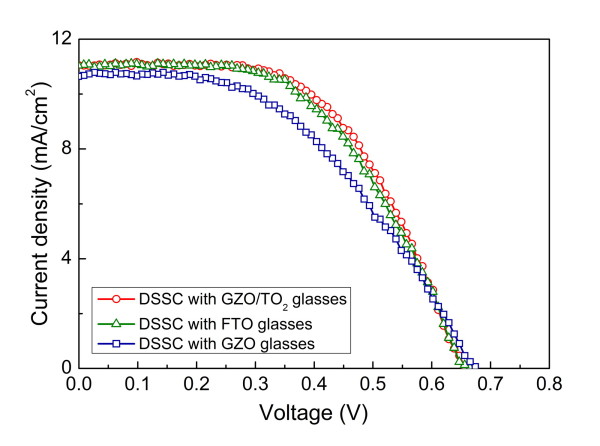
***J-V *characteristics of the fabricated DSSCs using the GZO/TiO_2_, GZO, and FTO glasses**.

Figure 4 shows the photocurrent density-voltage [*J-V*] characteristics of the fabricated DSSCs using the GZO/TiO_2_, GZO, and FTO glasses measured under one sun condition. The estimated photovoltaic parameters are summarized in Table 1. It should be noted that the performance of the DSSCs with the normal GZO glasses is relatively poor (3.36%) compared to that with the FTO though the GZO and FTO have similar electrical resistivity. However, when the TiO_2 _blocking layer is employed, the fabricated DSSCs show an improvement in the short-circuit current [*I*_sc_] and fill factor [FF], and as a result, a conversion efficiency of 4.02% is obtained, which is 19.6% higher than that of the DSSCs with the normal GZO glasses. These results prove that the TiO_2 _blocking layer plays a role in protecting the GZO films as was expected. Furthermore, the DSSCs with the GZO/TiO_2 _glasses show slightly better characteristics than those with the commercially used FTO glasses, which is ascribed to the fact that the TiO_2 _blocking layer can prohibit the recombination of injected electrons in the GZO with the electrolyte effectively. In particular, the improvement in FF is clearly found, which is due to the improved electrical contact between the GZO and TiO_2 _nanoparticles [9].

**Table 1:** Photovoltaic parameters of the fabricated DSSCs

Sample	*V*_oc _(V)	*J*_sc _(mA/cm^2^)	FF	*η *(%)
GZO/TiO_2_	0.652	11.05	0.558	4.021
GZO	0.673	10.64	0.47	3.363
FTO	0.656	11.04	0.531	3.844

**Figure 5:**
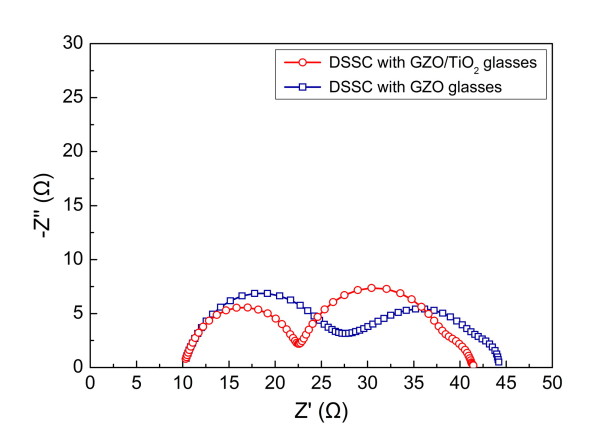
**Electrochemical impedance spectra of the fabricated DSSCs with and without the TiO_2 _blocking layer**.

Figure 5 presents the Nyquist plots of the electrochemical impedance spectra of the fabricated DSSCs with and without the TiO_2 _blocking layer. It is known that the semicircles in the frequency regions of 10^3 ^to 10^5^, 1 to 10^3^, and 0.1 to 1 Hz are associated with the charge transport at the TiO_2_/TCO or Pt/electrolyte interface, TiO_2_/dye/electrolyte interface, and Nernstian diffusion in the electrolyte, respectively [10]. The first circle of the DSSCs with the GZO/TiO_2 _glasses is smaller than that with the GZO glasses, which indicates that the charge transport at the TiO_2 _nanoparticles/GZO is easier with the TiO_2 _blocking layer. The larger shunt resistance of the DSSCs with the GZO/TiO_2 _glasses corresponding to the second circle is also seen. Therefore, the improvement in the efficiency with the TiO_2 _blocking layer can be explained by the small series resistance and large shunt resistance [11].

## Conclusions

GZO thin films were deposited on glass substrates for DSSC applications. The GZO films showed a low resistivity of 5.95 × 10^-4 ^Ω cm. A TiO_2 _blocking layer was inserted between the GZO and TiO_2 _nanoparticles to protect the GZO and enhance the photovoltaic properties. The fabricated DSSCs with the GZO/TiO_2 _glasses showed a conversion efficiency of 4.02%, which was 19.6% higher compared to 3.36% of the DSSCs using the GZO glasses. Furthermore, they showed slightly better characteristics than those using the commercially used FTO glasses. These results can be ascribed to the reduced charge recombination and series resistance caused by the influence of the TiO_2 _blocking layer. Therefore, it can be concluded that the GZO films have the ability to be employed to the high-efficiency DSSCs as transparent electrodes.

## Competing interests

The authors declare that they have no competing interests.

## Authors' contributions

JHK carried out the experiments and prepared the manuscript initially. KJL, JHR, and SWS participated in performing the experiments and characterizations. JHP and IHY participated in analyzing the experimental data. BMM conceived of the study and participated in its design and coordination. All authors read and approved the final manuscript.
